# Canine Cognitive Dysfunction from the Perspective of Dog Owners: Recognition, Care, and Emotional Challenges

**DOI:** 10.3390/ani16071117

**Published:** 2026-04-05

**Authors:** Viktória Balatonfüredi, Eniko Kubinyi

**Affiliations:** 1 Department of Ethology, ELTE Eötvös Loránd University, 1117 Budapest, Hungary; balatonfurediviktoria@gmail.com; 2 Doctoral School of Biology, Institute of Biology, ELTE Eötvös Loránd University, 1117 Budapest, Hungary; 3 MTA-ELTE Lendület “Momentum” Companion Animal Research Group, 1117 Budapest, Hungary; 4 ELTE NAP Canine Brain Research Group, 1117 Budapest, Hungary

**Keywords:** canine cognitive dysfunction, aging dogs, veterinary communication, caregiver burden, companion animal welfare

## Abstract

Canine cognitive dysfunction (CCD) is a progressive neurodegenerative condition affecting aging dogs, characterized by impairments in learning, memory, spatial orientation, and behavior. Despite its substantial negative impact on dogs’ quality of life and owners’ emotional well-being, CCD is frequently underrecognized or diagnosed at a late stage. This study explored how challenges in CCD recognition and veterinary communication influence dog owners’ ability to identify symptoms and make informed decisions about care. Semi-structured interviews were conducted with 22 dog owners whose dogs were suspected of having CCD, based on elevated scores on the Canine Cognitive Dysfunction Rating Scale (CCDR) and owner-reported behavioral changes. Interview data were analyzed using reflexive thematic analysis. Four main themes emerged: (1) difficulties in recognizing CCD-related symptoms, (2) communication challenges between owners and veterinarians, (3) owners’ adaptation to gradually emerging symptoms, and (4) the emotional and practical burden of caregiving. Owners frequently interpreted behavioral changes as normal aging or other health problems, which delayed the recognition of cognitive decline. Participants also described limited guidance from veterinary professionals regarding CCD, contributing to uncertainty, emotional distress, and challenges in end-of-life decision-making. Together, these findings suggest that owners’ experiences follow a progressive caregiving trajectory, from initial symptom uncertainty to increasing emotional and practical burden. Improving awareness of CCD, strengthening veterinary communication, and providing targeted support for caregivers may facilitate earlier recognition and more effective management of cognitive decline, ultimately benefiting both dogs and the people who care for them.

## Simple Summary

Canine cognitive dysfunction (CCD) is a dementia-like condition in older dogs that affects memory, behavior, and daily functioning. Although CCD can strongly reduce dogs’ quality of life and place a heavy emotional burden on owners, it is often recognized late or not at all. This study explored how dog owners experience CCD-like symptoms, how they understand these changes, and what challenges they face when seeking veterinary support. We conducted interviews with 22 dog owners whose dogs showed signs consistent with CCD. Owners described difficulties in recognizing symptoms, often interpreting them as normal aging or as other health problems. Many reported limited information and guidance from veterinarians, which increased uncertainty and emotional strain. As symptoms progressed, owners gradually adapted their daily routines and living environments while also experiencing exhaustion, anxiety, guilt, and grief related to end-of-life decisions. Our findings show that CCD-like symptoms affect not only the animals themselves but also the well-being of their caregivers. Improving early recognition, veterinary communication, and owner support could help dogs with CCD live more comfortably and reduce the emotional burden on owners.

## 1. Introduction

Advances in veterinary medicine, nutrition, and husbandry have substantially increased the lifespan of companion dogs over recent decades [1,2,3]. As a result, ensuring not only longevity but also a long healthspan, i.e., a good quality of life throughout the entire lifespan, has become a central concern in canine welfare research and clinical practice. Aging is a universal biological process involving the progressive decline of physiological functions necessary for survival and reproduction [4]. In dogs, healthy aging is increasingly conceptualized as the maintenance of physical, cognitive, and social well-being, supported by preventive veterinary care, appropriate environmental enrichment, and sustained social interaction [5,6,7,8,9,10].

Increased lifespan, however, is often accompanied by chronic degenerative diseases that compromise quality of life in later years. One such condition is canine cognitive dysfunction (CCD), also referred to as canine cognitive dysfunction syndrome (CCDS), a progressive neurodegenerative disorder affecting older dogs and characterized by impairments in learning, memory, spatial orientation, and behavior [1]. CCD leads to a marked deterioration in dogs’ vitality, social interactions, and daily functioning, and it can profoundly alter the dog–owner relationship, particularly when combined with sensory decline [11].

The clinical and pathological features of CCD show substantial similarities to human Alzheimer’s disease (AD). Affected dogs exhibit brain atrophy, neuronal loss, amyloid-β accumulation [12,13,14,15,16,17,18,19,20,21,22,23], neurofibrillary tangles, and alterations in neurotransmitter systems. In addition, emerging evidence suggests an association between memory decline and changes in gut microbiota composition, including an increased proportion of Actinobacteria, which may serve as a potential biomarker of AD [24]. The characteristic behavioral signs of CCD are commonly summarized by the DISHAA acronym (disorientation, altered interactions, sleep–wake cycle disturbances, house-soiling, changes in activity, and increased anxiety), often accompanied by cognitive, emotional, and physical impairments that closely parallel symptoms of human dementia [20,25,26]. These similarities make dogs a valuable natural model for studying age-related cognitive decline.

Despite its welfare relevance, CCD remains substantially underdiagnosed [27,28]. For example, in a U.S. marketing research summary, cited in [29], approximately 75% of owners of dogs aged seven years or older reported observing behavioral changes consistent with cognitive decline, yet only about 12% communicated these concerns to their veterinarian. In dogs older than eight years, the estimated prevalence of CCD is 14.2%, while the formal diagnosis rate is only 1.9% [27]. Recent survey data indicate that although most veterinarians are familiar with the concept of CCD, many diagnose only a small number of cases annually, suggesting that symptoms are frequently interpreted as part of normal aging rather than as indicators of a neurodegenerative condition [30].

In clinical practice, the diagnosis of canine cognitive dysfunction is typically based on a combination of owner-reported behavioral changes, clinical examination, and the exclusion of other medical conditions that may present with similar signs [1]. Structured screening tools and questionnaires may support the recognition of cognitive decline; however, these approaches rely heavily on subjective interpretation and retrospective reporting by owners. This reliance can contribute to missed or delayed diagnosis, particularly in the early stages when behavioral changes are subtle, inconsistent, or attributed to normal aging. Furthermore, the absence of validated and routinely available diagnostic biomarkers in veterinary practice limits the ability to establish a more objective diagnosis, reinforcing the importance of owner observations while simultaneously introducing uncertainty into the diagnostic process.

The under-recognition of CCD has important implications not only for affected dogs, but also for their owners. The human–dog relationship often develops into a deep emotional bond, with dogs frequently perceived as family members and sources of emotional support, particularly for individuals living alone [31]. Consequently, the onset of cognitive decline in a dog can place a substantial emotional burden on owners, including feelings of worry, helplessness, and anticipatory grief [32]. In addition, the long-term care of elderly or cognitively impaired dogs may entail considerable financial costs related to veterinary care, medication, and assistive devices, which can further strain owners’ resources and well-being [33].

Although questionnaire-based studies have contributed valuable epidemiological data on CCD, relatively little is known about how owners subjectively experience their dog’s cognitive decline. Research focusing on owners’ lived experiences, decision-making processes, and interactions with veterinary professionals in the context of CCD is largely lacking. Qualitative approaches are especially well-suited to capturing these complex emotional, practical, and relational dimensions, which are often overlooked in quantitative research.

The present study aims to address this gap by exploring, through in-depth semi-structured interviews with dog owners, how CCD-related behavioral changes are perceived and interpreted, what emotional and practical challenges owners face, and how they experience uncertainty, limited information, and perceived lack of support during the progression of the disease. By foregrounding the owners’ perspective, this research seeks to contribute to a more comprehensive understanding of CCD that can inform future diagnostic practices, veterinary education, and support systems, improving the welfare of both dogs and their caregivers.

## 2. Materials and Methods

### 2.1. Ethics

The study was approved by the Institutional Review Board (protocol code No. 2022-62, approved on 21 April 2022). Prior to participation, all owners received written and verbal information about the purpose of the study, the voluntary nature of participation, procedures for data handling, and confidentiality. Written informed consent was obtained from all participants before data collection.

### 2.2. Participants

A total of 22 dog owners participated in the study, aged between 28 and 64 years. Most participants resided in urban areas (13 in the capital city Budapest, 7 in other cities, and 2 in villages). Thirteen participants lived in apartments and nine in detached houses. Educational attainment and labor market status largely reflected a highly educated sample (Table 1). All participants who volunteered and met the inclusion criteria were women. To ensure anonymity, all participants were assigned identification numbers, which were used throughout the manuscript and in Table 1.

The dogs’ age ranged from 12.5 to 20.5 years, all were neutered, and 13 were females. Eleven dogs were mixed-breed, and body weights ranged from 3.5 to 24 kg. CCDR scores ranged from 41 to 78; 5 dogs fell into the mild impairment category (40–49 points), and 17 met the criterion for suspected CCD (at least 50 points; Table 1).

### 2.3. Procedure

Participants were recruited through multiple channels, including a public call on social media, personal contacts, and recommendations. In the initial phase, dog owners completed an online version of the Canine Cognitive Dysfunction Rating (CCDR) questionnaire [34], which was extended with demographic items and distributed via relevant social media groups.

Participants whose dogs scored at least 40 points on the CCDR scale and who indicated a willingness to participate in further studies were contacted individually, and interview appointments were arranged by telephone. Dogs were classified as having suspected CCD based on owner-observed behavioral changes consistent with CCD symptoms rather than on a formal veterinary diagnosis. A clinical diagnosis was not required for inclusion, as the study aimed to capture the owners’ lived experiences during a phase in which cognitive decline may be perceived but not yet clinically confirmed, rather than relying on formally diagnosed cases. This approach reflects the reality of underdiagnosis and the delayed recognition of CCD in veterinary practice.

The CCDR [34] is designed to capture signs observable in everyday situations. Symptoms are commonly organized according to the DISHAA categories (disorientation, changes in social interaction, sleep–wake disturbances, house soiling, activity, anxiety). Based on the total CCDR score, three classifications are used: 0–39 points = normal cognitive status (non-CCD), 40–49 points = at risk (mild impairment; “pre-CCD”), and ≥50 points = suspected CCD.

Semi-structured interviews were conducted between March 2023 and March 2025 by the first author (VB) in Hungarian, following a reflexive thematic analysis approach [35]. Data collection continued until sufficient information power was reached to address the study aims and support robust theme development. While the interview guide remained consistent throughout data collection, interviews were conducted flexibly, allowing participants to elaborate on their experiences and introduce emerging topics. Iteration occurred primarily during the analytical process through repeated reading of the data, coding, and refinement of themes.

Fourteen interviews were conducted online via video conferencing platforms, seven in person, and one via written email correspondence. The interview guide was developed based on questions from previous studies on aging and health in dogs [33,35], and was adapted to align with the aims of the present research.

The interview guide covered the following topics: owners’ first observations of behavioral changes, interpretations of these changes, experiences with veterinary consultations, emotional responses and coping strategies, practical caregiving challenges, decision-making regarding treatment and end-of-life considerations, and perceived availability of professional and social support. Interviews lasted approximately 40–60 min, were audio-recorded, and transcribed verbatim. Coding and analysis were conducted manually by the researchers; this approach facilitated close, iterative engagement with the data, which is consistent with reflexive thematic analysis and does not require the use of qualitative data analysis software. Translations from Hungarian into English were verified using a back-translation procedure.

### 2.4. Data Analysis

Interview transcripts were analyzed using reflexive thematic analysis, following the approach described by Braun et al. [36]. This method was chosen for its theoretical flexibility and suitability for identifying patterned meanings across qualitative data while remaining sensitive to the participants’ subjective experiences.

Analysis proceeded through several phases. First, the researchers familiarized themselves with the data through repeated reading of the transcripts and initial discussions of emerging ideas. Second, inductive, semantic-level codes were generated to capture salient meanings and experiences expressed by the participants. During this phase, coding remained close to the participants’ original wording to preserve nuance and contextual richness.

Although reflexive thematic analysis does not aim to establish inter-rater reliability, both authors were actively involved in coding and theme development to support reflexive engagement with the data. Differences in interpretation were discussed collaboratively, leading to the refinement of code definitions and thematic organization.

In the final analytic phase, conceptually related codes were clustered into four overarching themes that reflected the main dimensions of owners’ experiences:

(1) Difficulties in recognizing CCD-related symptoms;
(2) Communication challenges between owners and veterinarians;
(3) The gradual onset of CCD-related symptoms and owners’ adaptation to these changes;
(4) The emotional and practical burden of caring for a dog with suspected CCD.

Both spontaneous narratives and responses to guiding questions were included in the analysis. Relevant verbatim excerpts were highlighted and accompanied by brief analytic summaries to capture their contextual meaning. Individual segments of text could be assigned to multiple codes, reflecting the overlapping and interconnected nature of the owners’ experiences.

## 3. Results

The thematic analysis identified four themes reflecting a progressive trajectory in owners’ experiences with canine cognitive dysfunction from initial symptom recognition to caregiving burden and end-of-life decision-making. Participants’ accounts described a process beginning with difficulties in recognizing early symptoms, followed by attempts to seek veterinary guidance. As cognitive decline progressed, owners gradually adapted their home environments and daily routines to meet their dogs’ changing needs. Over time, these adaptations often resulted in increasing emotional and practical burdens, including exhaustion, anticipatory grief, and complex end-of-life decision-making. The themes and codes are summarized in Table 2.

Across the 22 interviews, owners’ adaptation to progressive symptoms emerged in all interviews, reflected in accounts of gradual functional decline, increasing nighttime caregiving demands, and ongoing adjustments to the home environment.

Recognition of CCD-related symptoms was also highly frequent and was reported by all but one participant; owners described normalization of early signs, diagnostic uncertainty, retrospective recognition, and the gradual accumulation of symptoms over time.

The emotional and practical burden of caregiving was present in 19 interviews, encompassing emotional distress, restrictions on daily life, changes in the owner–dog relationship, and ethical dilemmas related to end-of-life decisions.

Finally, owner–veterinarian communication appeared in 18 interviews, including both negative experiences—such as perceived lack of knowledge, insufficient guidance, and limited emotional support—and more positive, supportive interactions.

### 3.1. Recognizing CCD-Related Symptoms

Owners frequently described substantial challenges in recognizing the early signs of suspected canine cognitive dysfunction (CCD). Within this theme, four interrelated codes were identified: normalization of symptoms, diagnostic uncertainty, retrospective recognition, and symptom accumulation.

Many participants initially interpreted behavioral changes as part of normal aging rather than as potential indicators of a medical condition, reflecting the code *normalization of symptoms*. Several owners reported that they had never previously encountered the concept of dementia in dogs. As one participant explained, *“Honestly, dog dementia was completely new to me”* (O10). In this context, subtle behavioral changes such as disorientation, altered sleep patterns, or changes in interaction were often normalized and therefore not initially perceived as signs of cognitive decline.

Even when owners noticed unusual behaviors, many expressed uncertainty about their underlying causes, corresponding to the code *diagnostic uncertainty*. Participants frequently struggled to determine whether the observed changes reflected cognitive decline or other age-related conditions, such as sensory impairment or chronic disease. For example, one owner explained: *“It’s hard to tell which symptoms came from Cushing’s disease and which from dementia”* (O20). Similarly, another participant described difficulties distinguishing cognitive decline from vision problems: *“I don’t know whether this is dementia or vision loss—I really don’t know”* (O5). In some cases, hearing impairment further complicated interpretation, making it difficult for owners to attribute behavioral changes to a specific underlying cause.

In several cases, recognition occurred only after owners encountered structured information about CCD, which is reflected in the code *retrospective recognition*. Some participants reported that completing a symptom questionnaire or reading about CCD prompted them to reconsider earlier behavioral changes. One owner described this experience after seeing the CCDR questionnaire: *“When I saw the questionnaire—I thought, my God, almost everything fits exactly what was written there”* (O6). Similarly, another participant noted that early signs became meaningful only in hindsight: *“Back then I didn’t notice dementia as such… but that’s where something started”* (O4).

Other participants described a gradual emergence of multiple behavioral changes over time, captured by the code *symptom accumulation*. Initially isolated or infrequent symptoms became more frequent or were joined by additional changes, eventually forming a pattern that owners could recognize as problematic. As one owner explained: *“At first it was extremely rare… but then slowly more signs appeared”* (O8).

Taken together, these accounts suggest that the recognition of suspected CCD is often delayed due to the limited awareness of canine dementia, the gradual onset of symptoms, and the overlap between cognitive decline and other age-related health problems. As a result, behavioral changes were frequently normalized or only recognized retrospectively once symptoms had accumulated or when owners encountered more structured information about the condition.

### 3.2. Owner–Veterinarian Communication

Many participants described gaps in communication between themselves and veterinary professionals regarding the recognition and management of CCD. Within this theme, four codes were identified: perceived lack of veterinary knowledge or recognition of CCD, insufficient guidance and practical advice, emotional and communicative gaps in consultations, and positive experiences of supportive veterinary communication.

Several owners felt that veterinarians did not recognize or sufficiently consider cognitive decline as a potential explanation for their dogs’ behavioral changes, corresponding to the code *perceived lack of veterinary knowledge or recognition of CCD*. One participant noted that the symptoms were not considered together as part of a cognitive syndrome: *“The veterinarian didn’t really put the symptoms together and consider that it might be dementia”* (O16). Some owners also expressed general dissatisfaction with previous veterinary encounters, describing negative experiences that affected their confidence in professional guidance. As one owner summarized: *“I’ve had quite a few bad experiences”* (O22). Another participant noted that although some positive encounters occurred, overall dissatisfaction remained: *“There were positive ones too, but the negatives outweighed them”* (O2).

Participants also frequently described receiving limited practical advice on how to manage the daily challenges associated with caring for an aging dog with suspected cognitive decline. This pattern is reflected in the code *insufficient guidance and practical advice*. Several owners reported that they had to seek information independently through online sources or personal networks because they did not receive sufficient guidance during veterinary consultations. One owner expressed a desire for more detailed discussions and practical recommendations: *“I would really appreciate talking it through and getting advice”* (O1). The same participant emphasized the absence of concrete information on how to manage everyday life with an elderly dog: *“I have no information on how to live with an old dog”* (O1).

In addition to informational gaps, some owners perceived a lack of emotional support or engagement during consultations, corresponding to the code *emotional and communicative gaps in consultations*. Participants reported that veterinary visits often focused primarily on physical health indicators, while cognitive changes and the emotional challenges associated with caring for a declining pet received less attention. For example, one owner described the veterinarian’s approach as limited to basic physical functioning: *“The vet says that as long as she eats and drinks, she’s fine… and that when she becomes paralyzed, then we’ll have to decide how she should go on. That’s usually all he says”* (O1). Some participants also perceived disparities in the quality of veterinary care between different regions, particularly between urban and rural settings. One participant commented critically: *“Veterinarians in rural areas are terribly behind compared to human medicine”* (O13).

At the same time, not all experiences were negative. Several owners described highly supportive relationships with their veterinarians, captured in the code *positive experiences of supportive veterinary communication*. In these cases, participants emphasized empathy, openness, and the opportunity to discuss concerns in detail. As one owner explained: *“You can talk through everything with him… we really like going there”* (O4). Such experiences highlighted the value of attentive communication and collaborative decision-making when managing complex age-related conditions.

Overall, the owners’ accounts revealed considerable variability in veterinary communication regarding CCD. While some participants described supportive and informative interactions, others perceived indifference, limited knowledge, or insufficient guidance. Many owners reported a lack of discussion about cognitive symptoms and practical caregiving strategies, which contributed to uncertainty and a sense of being left alone with difficult decisions related to their dogs’ care and well-being.

### 3.3. Owners’ Adaptation to Progressive Symptoms

Participants frequently described the progressive nature of suspected CCD and the gradual adjustments they made in response to their dogs’ changing needs. Within this theme, four codes captured these experiences: gradual functional decline, incremental adaptation of the home environment, nighttime caregiving demands, and normalization of increasing caregiving burden.

Many owners first noticed a slow decline in their dogs’ previously well-established abilities, corresponding to the code *gradual functional decline*. Participants described how behaviors that had once been routine gradually became more difficult for their dogs. One owner reflected on this process by noting: *“The abilities that used to work well slowly started to fade”* (O9). Because these changes emerged gradually, they were often perceived as part of a natural aging process rather than as symptoms of a specific cognitive disorder (see also *normalization of symptoms* in Theme 1).

As their dogs’ functional abilities declined, owners frequently modified their living environments to maintain safety and accessibility, reflecting the code *incremental adaptation of the home environment*. Participants described adjusting furniture arrangements or restricting access to certain areas of the home to prevent disorientation or accidents. One owner explained that the height of the dog’s bed had been lowered to accommodate reduced mobility: *“You can see how low the bed is—that’s because she can’t jump up anymore”* (O10). Similarly, another participant described creating physical barriers within the home to prevent the dog from becoming trapped: *“We had to fence off part of the apartment so she wouldn’t get stuck”* (O12).

Nighttime disorientation was frequently mentioned as a particularly demanding aspect of caregiving, corresponding to the code *nighttime caregiving demands*. Several participants reported that their dogs became restless, confused, or trapped in corners during the night, requiring repeated intervention. As one owner described: *“Her dementia only mildly affects daily life, but we get up two or three times every night to help her out of corners or stop her from circling”* (O18). Another participant described the repetitive nature of these nighttime interruptions: *“I get up half-asleep and put her back in place”* (O9). Such disruptions required constant vigilance and often resulted in significant sleep deprivation for owners.

Over time, these incremental adjustments contributed to a broader pattern captured in the code *normalization of increasing caregiving burden*. Because symptoms and caregiving demands developed gradually, owners often adapted step by step to new routines without immediately recognizing the cumulative impact on their own well-being. The progressive nature of CCD meant that changes were integrated into everyday life as they emerged, which reduced the perceived urgency of seeking external assistance. As caregiving responsibilities increased—particularly during nighttime—owners described experiencing both physical exhaustion and emotional strain.

Overall, the participants’ accounts illustrate how the gradual onset of suspected CCD symptoms shaped both recognition and caregiving practices. The slow progression of behavioral and functional changes encouraged incremental adaptations in the home environment and daily routines while simultaneously normalizing a growing caregiving burden that often went unrecognized until symptoms had become more pronounced.

### 3.4. Emotional and Practical Burden of Caregiving

Participants frequently described the emotional and practical difficulties associated with caring for dogs experiencing cognitive decline. Within this theme, four codes captured these experiences: emotional distress associated with cognitive decline, restrictions on owners’ daily life and exhaustion, personality changes and altered relational connection, and guilt and ethical uncertainty in end-of-life decision-making.

Many owners described profound emotional distress while observing their dogs’ cognitive and relational decline, corresponding to the code *emotional distress associated with cognitive decline*. Participants often experienced the gradual loss of familiar forms of interaction and communication with their dogs. One owner expressed this sense of loss by stating: *“I feel like I’m starting to lose communication with her; her world is closing in. It’s suffocating”* (O1). Others described persistent anxiety related to their dogs’ condition, particularly when they were not physically present to monitor them. As one participant explained, returning home could be emotionally charged due to uncertainty about the dog’s condition: *“We come in afraid every time—alive or not”* (O5).

In addition to emotional distress, caregiving responsibilities often imposed substantial constraints on owners’ daily lives, reflected in the code *restrictions on owners’ daily life and exhaustion*. Participants described how the increasing needs of their dogs limited their personal freedom and required constant attention. One owner summarized this experience as follows: *“I feel very tied down, and the freedom we used to have is gone”* (O16). Another participant described the cumulative physical strain associated with caregiving: *“It limits my life so much… I’m extremely tired”* (O3). Such demands frequently reduced the time owners had for their own activities or rest.

Some participants also described noticeable changes in their dogs’ personalities, corresponding to the code *personality changes and altered relational connection*. Owners observed that behaviors and traits that had previously defined their dogs’ character gradually disappeared as cognitive decline progressed. One owner reflected on this difficult process by noting: *“Her personality is starting to disappear—and that’s what makes it a bit easier”* (O17). These experiences often reshaped the emotional relationship between owners and their dogs, contributing to complex feelings of loss and adjustment.

Finally, many owners described feelings of guilt and ethical uncertainty when confronting end-of-life decisions, captured in the code *guilt and ethical uncertainty in end-of-life decision-making*. Participants struggled to balance the desire to prolong their dogs’ lives with concerns about their quality of life and dignity. One owner articulated this tension by stating: *“I want to let her go with dignity… and of course, I feel guilty”* (O1). Others described the emotional weight of anticipating the inevitable farewell while continuing to provide intensive care: *“This is a very sensitive topic for us now, because we’ll have to let her go soon. We’re doing everything we can for her, even though we have almost no time for ourselves”* (O18).

Taken together, these accounts highlight the substantial emotional and practical burden associated with caring for dogs with suspected CCD. Participants described exhaustion, anxiety, guilt, and anticipatory grief, often intensified by uncertainty surrounding end-of-life decisions and the limited social recognition of pet-related loss. These findings indicate that suspected CCD affects not only canine welfare but also the mental well-being of caregivers, underscoring the importance of supportive frameworks that address both members of the human–dog relationship.

## 4. Discussion

The aim of the present study was to explore how dog owners perceive and interpret early and advanced signs of suspected canine cognitive dysfunction (CCD), how they experience veterinary communication and care, and how they cope with the emotional and practical burden associated with caring for an aging dog with cognitive decline. By focusing on owners’ lived experiences, this qualitative study provides insight into processes that precede clinical recognition and shape caregiving trajectories in everyday life. The findings suggest that owners’ experiences of suspected CCD follow a progressive caregiving trajectory, beginning with symptom uncertainty, followed by help-seeking and adaptation, and eventually leading to increasing emotional and practical caregiving burden. These caregiving experiences show notable conceptual parallels with those described in human neurodegenerative conditions such as Alzheimer’s disease, particularly in relation to emotional strain, disruption of daily routines, and anticipatory grief.

Consistent with previous research, owners in our study described suspected CCD symptoms as emerging gradually rather than appearing suddenly [37]. This slow progression made early recognition particularly challenging and often led owners to interpret behavioral changes as part of normal aging rather than as signs of a pathological process, in line with earlier findings [29,35]. Owners’ accounts illustrate how the overlap between suspected CCD symptoms and other age-related conditions—such as sensory decline, chronic pain, or endocrine disorders—contributed to uncertainty and delayed interpretation. This reflects the known clinical heterogeneity of CCD [38] and the fact that diagnosis relies largely on exclusion through differential diagnostic procedures [1].

The gradual onset of symptoms not only hindered recognition but also shaped owners’ adaptive responses. Many participants described progressively modifying their living environment and daily routines to accommodate their dogs’ changing needs, such as setting up barriers at night, adjusting sleeping arrangements, or increasing supervision. While such adaptations may enhance short-term safety and comfort, they may also reduce the perceived urgency of seeking professional support, particularly when owners believe that they can manage symptoms at home. This finding aligns with reports that owners are less likely to consult veterinarians when behavioral changes develop slowly or resemble previously encountered age-related issues [37].

Veterinary communication emerged as a critical factor influencing owners’ experiences. Several participants reported insufficient guidance, limited discussion of cognitive symptoms, or a perceived lack of empathy, which contributed to feelings of uncertainty and isolation. Ineffective communication poses a barrier to care in two ways: owners may fail to report relevant symptoms or adhere to recommendations if they do not fully understand their significance, and veterinarians may miss opportunities to collaboratively develop tailored care plans if owners’ observations and concerns are not fully explored. These findings highlight the need for improved veterinary communication practices characterized by active listening, clear explanations, empathy, and personalized guidance. In the Hungarian context, the limited availability or visibility of veterinary expertise specifically focused on cognitive decline may further contribute to the inconsistent recognition and prioritization of CCD in general practice, reinforcing the importance of continuing education and structured informational resources.

Beyond practical caregiving challenges, owners described profound emotional distress associated with their dogs’ cognitive decline. Given the strength of the human–dog bond and the frequent perception of dogs as family members or primary emotional companions, witnessing cognitive and personality changes was experienced as deeply painful. Many owners reported feelings consistent with anticipatory grief—mourning the gradual loss of their dog before death occurs [39]. This grief was often intensified by uncertainty surrounding euthanasia decisions and by the moral responsibility owners felt in determining the appropriate time to let their dog go.

The emotional burden described by participants aligns with findings by Silva et al. [32], who emphasize that well-informed, empathetic veterinary support can mitigate feelings of guilt, anger, and the risk of prolonged grief. However, owners in the present study frequently felt that such support was limited or unavailable. Cordaro [40] further notes that grief related to pet loss is often socially unrecognized, leading to disenfranchised grief and increased loneliness. These observations suggest that support for owners of dogs with suspected CCD should extend beyond medical management to include psychological and community-based resources, such as counseling services or peer support groups, that acknowledge the legitimacy of pet-related grief.

This study has several limitations that should be considered when interpreting the findings. First, the sample size was relatively small and predominantly urban and highly educated, which may limit the transferability of the results to other populations of dog owners, particularly those in rural areas or with different socioeconomic backgrounds. Second, dogs were classified as having suspected CCD based on owner reports and CCDR scores rather than formal veterinary diagnoses; while this approach was appropriate for capturing owners’ lived experiences in the context of underdiagnosis, it may have included dogs with other age-related conditions presenting similar symptoms. Third, data were collected using multiple interview modalities (online, in-person, and written email), which may have influenced the depth and richness of some narratives. Finally, as with all qualitative research, the findings are interpretive and context-dependent and do not aim to provide prevalence estimates or causal inferences. Despite these limitations, the study offers valuable insight into owner experiences that are difficult to capture using quantitative methods alone and highlights important areas for future research and clinical practice.

Looking forward, advances in biological markers may significantly improve the early detection and monitoring of CCD. Recent studies suggest that blood-based biomarkers, including neurofilament light chain (NfL), retinol-binding protein 4 (RBP4), CXCL10, and NOX4, may reflect underlying neurodegenerative and inflammatory processes and hold promise as diagnostic tools [41,42]. In parallel, the development of standardized behavioral tests targeting cognitive indicators associated with CCD severity may further enhance early recognition and longitudinal assessment. However, until such tools become widely accessible in clinical practice, the identification of cognitive decline will continue to rely heavily on detailed owner observations, regular veterinary visits, and effective, empathetic communication.

Overall, our findings underscore that CCD is not only a veterinary and welfare issue but also a relational and emotional challenge that affects the entire human–dog dyad. These findings also align with a One Health perspective, highlighting how the health and well-being of aging companion animals are closely intertwined with the psychological well-being of their caregivers. Improving awareness, communication, and support structures for veterinarians and owners alike may facilitate earlier recognition, more informed decision-making, and better quality of life for both dogs with CCD and the people who care for them. Understanding this caregiving trajectory may help veterinarians identify critical points at which targeted communication and support could reduce uncertainty and caregiver burden.

## 5. Conclusions

This study highlights that CCD-related symptoms are frequently under-recognized by owners due to their gradual onset, symptom overlap with normal aging, and limitations in veterinary communication and support. Owners play a central role in identifying early behavioral changes, yet they often face uncertainty, emotional burden, and a lack of guidance during the progression of the disease. Early, proactive screening, more standardized diagnostic approaches to exclude other age-related conditions, structured and empathetic communication, and owner-tailored support strategies and protocols might together improve recognition, enhance dogs’ quality of life, and promote owners’ well-being. Addressing CCD therefore requires an integrated approach that acknowledges both the clinical and relational dimensions of aging in companion dogs.

## Figures and Tables

**Table 1:** Characteristics of the participants included in the study (*: age estimated by VB).

Owner ID	Age (Years)	Location	Housing Type	Educational Attainment	Employment Status	Dog Age (Years)	Breed	Sex of Dog (All Neutered)	CCDR Score	Dog Body Weight (kg)
O1	30	Capital city	Detached house	High school diploma	Police officer	16	Wire-haired dachshund	Male	77	12
O2	35 *	Capital city	Apartment	College/University degree	Not reported	14	Fox terrier	Female	78	7
O3	46	Capital city	Apartment	College/University degree	Project coordinator	18	Bichon Havanese	Female	61	4.3
O4	64	Village	Detached house	College/University degree	Special education teacher	17	Shih-tzu	Male	58	3.5
O5	58	Village	Detached house	High school diploma	Nurse	16	Labrador retriever	Female	46	24
O6	29	Capital city	Apartment	College/University degree	Mental health professional	18	Mixed breed	Female	72	13
O7	35	Capital city	Apartment	College/University degree	Executive assistant	18	Pug	Male	55	12.6
O8	48	City	Detached house	High school diploma	Collections specialist	16	Beagle	Male	57	22
O9	59	Capital city	Apartment	College/University degree	Teacher	17	Mixed breed	Female	76	6.5
O10	52	Capital city	Apartment	College/University degree	Pediatric medical assistant	15	Mixed breed	Male	67	14
O11	37	Capital city	Apartment	College/University degree	Veterinarian	17	Miniature Pinscher	Male	41	6.5
O12	62	City	Detached house	College/University degree	Boarding school educator	20.5	Bichon Havanese	Female	57	4
O13	42	City	Detached house	College/University degree	Physician	14	Border collie	Female	56	19
O14	31	City	Detached house	College/University degree	Small animal physiotherapist	15	Mixed breed	Male	49	19
O15	35	City	Detached house	Post-secondary vocational education	Canine physiotherapist	16	Mixed breed	Female	46	9
O16	38	City	Detached house	College/University degree	Tax advisor	14	Mixed breed	Male	54	10.5
O17	33	Capital city	Apartment	Post-secondary vocational education	Trainer—FinTech customer support	15	Mixed breed	Female	58	12
O18	48	City	Apartment	High school diploma	Self-employed (manicurist/pedicurist)	18	Mixed breed	Male	65	10.5
O19	52	Capital city	Apartment	College/University degree	Special education teacher	12.5	Mixed breed	Female	46	20.3
O20	28	Capital city	Apartment	College/University degree	Analyst	15	Mixed breed	Female	72	11.8
O21	49	Capital city	Apartment	College/University degree	Psychologist	16.5	Mixed breed	Female	61	6.5
O22	64	Capital city	Apartment	Post-secondary vocational education	Assistant	16	Border collie	Female	51	13

**Table 2:** Themes and codes identified by the thematic analysis.

Theme	Code	Description
Recognizing CCD-related symptoms	Normalization of symptoms	Behavioral changes interpreted as normal aging
	Diagnostic uncertainty	Difficulty distinguishing CCD from other age-related conditions or sensory impairments
	Retrospective recognition	Earlier behavioral changes recognized as potential CCD signs only in hindsight
	Symptom accumulation	Recognition emerging as multiple behavioral changes gradually formed a noticeable pattern
Owner–veterinarian communication	Perceived lack of veterinary knowledge or recognition of CCD	Owners felt veterinarians did not sufficiently recognize or address cognitive decline
	Insufficient guidance and practical advice	Limited information about managing CCD or caring for an aging dog
	Emotional and communicative gaps in consultations	Veterinary interactions perceived as focused mainly on physical health with limited emotional support
	Positive experiences of supportive veterinary communication	Empathic, attentive consultations characterized by discussion and collaborative decision-making
Owners’ adaptation to progressive symptoms	Gradual functional decline	Progressive loss of previously established abilities and behaviors
	Incremental adaptation of the home environment	Modifying living spaces to maintain safety and accessibility for the dog
	Nighttime caregiving demands	Managing nocturnal disorientation, wandering, or circling requiring repeated intervention
	Normalization of increasing caregiving burden	Gradual adaptation leading owners to accept growing caregiving demands as part of daily life
Emotional and practical burden of caregiving	Emotional distress associated with cognitive decline	Emotional pain related to observing cognitive and relational decline
	Restrictions on owners’ daily life and exhaustion	Caregiving responsibilities limiting personal freedom and causing fatigue
	Personality changes and altered relational connection	Perceived loss of the dog’s former personality and changes in the human–dog relationship
	Guilt and ethical uncertainty in end-of-life decision-making	Moral tension and guilt surrounding euthanasia and quality-of-life decisions

## Data Availability

The qualitative interview data supporting the findings of this study are available from the corresponding author upon reasonable request.
